# Diagnosis and Nursing Intervention of Gynecological Ovarian Endometriosis with Magnetic Resonance Imaging under Artificial Intelligence Algorithm

**DOI:** 10.1155/2022/3123310

**Published:** 2022-06-11

**Authors:** Nijie Jiang, Hong Xie, Jiao Lin, Yun Wang, Yanan Yin

**Affiliations:** ^1^Department of Gynecology, West China Second University Hospital, Chengdu 610041, Sichuan, China; ^2^Department of Radiology, West China Second University Hospital, Chengdu 610041, Sichuan, China

## Abstract

This research was aimed to study the application value of the magnetic resonance imaging (MRI) diagnosis under artificial intelligence algorithms and the effect of nursing intervention on patients with gynecological ovarian endometriosis. 116 patients with ovarian endometriosis were randomly divided into a control group (routine nursing) and an experimental group (comprehensive nursing), with 58 cases in each group. The artificial intelligence fuzzy C-means (FCM) clustering algorithm was proposed and used in the MRI diagnosis of ovarian endometriosis. The application value of the FCM algorithm was evaluated through the accuracy, Dice, sensitivity, and specificity of the imaging diagnosis, and the nursing satisfaction and the incidence of adverse reactions were used to evaluate the effect of nursing intervention. The results showed that, compared with the traditional hard C-means (HCM) algorithm, the artificial intelligence FCM algorithm gave a significantly higher partition coefficient, and its partition entropy and running time were significantly reduced, with significant differences (*P* < 0.05). The average values of Dice, sensitivity, and specificity of patients' MRI images were 0.77, 0.73, and 0.72, respectively, which were processed by the traditional HCM algorithm, while those values obtained by the improved artificial intelligence FCM algorithm were 0.92, 0.90, and 0.93, respectively; all the values were significantly improved (*P* < 0.05). In addition, the accuracy of MRI diagnosis based on the artificial intelligence FCM algorithm was 94.32 ± 3.05%, which was significantly higher than the 81.39 ± 3.11% under the HCM algorithm (*P* < 0.05). The overall nursing satisfaction of the experimental group was 96.5%, which was significantly better than the 87.9% of the control group (*P* < 0.05). The incidence of postoperative adverse reactions in the experimental group (7.9%) was markedly lower than that in the control group (24.1%), with a significant difference (*P* < 0.05). In short, MRI images under the artificial intelligence FCM algorithm could greatly improve the clinical diagnosis of ovarian endometriosis, and the comprehensive nursing intervention would also improve the prognosis and recovery of patients.

## 1. Introduction

Endometriosis is a disease caused by the growth of the endometrium from its normal position. Endometrial cells are originally on the intrauterine mucosa and undergo periodic changes under the control of estrogen and progesterone [1]. Endometrial cells may fall off regularly and be excreted in the menstrual blood and may also become a breeding ground for the development of fertilized eggs. However, part of the “rebellious” endometrium escaped from the uterine cavity and “camped” in the pelvic peritoneum, ovaries, uterine surface, uterosacral ligament, and so on, causing a series of symptoms [2]. According to the involved positions, endometriosis can be classified into ovarian, peritoneal, deep infiltrating, and other parts of endometrioses [3]. It can invade any part of the body, most of which are in the pelvic organs and parietal peritoneum; ovaries and fundus ligaments are the most common position of endometriosis, followed by the uterus, visceral peritoneum, vaginal rectum, etc.; sometimes, it also occurs in the umbilicus, bladder, kidney, ureter, lung, pleura, breast, and even arms and thighs [4]. Endometriosis has a certain familial aggregation, and it is common in women of childbearing age. The incidence is 20%–90% in patients with chronic pelvic pain and dysmenorrhea, 25%–35% of infertility patients are related to endometriosis, and about 5%–15% of patients are accidentally found to have endometriosis during surgery [5–7]. At present, there is no method to cure this disease completely, but it can be relieved and controlled by medication and surgical treatment. Therefore, timely and effective screening and diagnosis are very important for the clinical control of endometriosis.

Nowadays, clinical examination methods for endometriosis include gynecological examination, laboratory examination, and imaging examination. The imaging examination methods mainly include B-scan ultrasonography, pelvic computed tomography (CT), and plain scan of magnetic resonance imaging (MRI) [8]. Among these imaging diagnostic techniques, ultrasound examination is often used for endometriosis screening because of its fast convenience and low cost. CT has a high-density resolution and can display organs and soft tissue structures with small density differences clearly, but it is unable to judge the relationship between the mass and the surrounding organs [9, 10]. Because of the advantages of multiparameter and multisequence imaging and high tissue resolution, a plain scan of MRI is very suitable for the diagnosis and staging of deep infiltrating endometriosis. It helps a lot to find vaginal rectal septum or rectal mass and identify the relationship between the mass and the surrounding organs like the intestine and bladder clearly, but there are still limitations in image noise and image detail imaging [11]. In addition, traditional MRI diagnosis often requires a comprehensive diagnosis by professional physicians, with effective information in the images and various characteristics of the patients. Such a process requires a high level of experience for the doctor. Medical imaging segmentation technology based on computer-assisted artificial intelligence algorithms has been developed in recent years, and it can restore the patient's image data to the largest extent and reduce the difficulty of identification for doctors [12].

The reported artificial intelligence-based medical image segmentation methods include convolutional neural network algorithm, random forest algorithm, clustering algorithm, and U-shaped network algorithm. These algorithms have their own advantages and disadvantages [13, 14]. At current, there is no report on artificial intelligence algorithms for MRI images of ovarian endometriosis. In addition, clinical studies have shown that comprehensive nursing can well assist surgical treatment. It can speed up the recovery process of patients, reduce the incidence of postoperative complications, improve the satisfaction of patients with treatment, and achieve better recovery effects.

Therefore, it was expected to design an artificial intelligence fuzzy C-means (FCM) clustering algorithm for the MRI image characteristics of patients with ovarian endometriosis and apply it to clinical MRI diagnosis. The recovery of ovarian endometriosis patients under different nursing methods was compared to evaluate the practical value of comprehensive nursing in the ovarian endometriosis treatment. Meanwhile, the performance and the diagnostic accuracy of the artificial intelligence algorithm were adopted for comprehensive evaluation of the application potential of the algorithm. It was hoped to provide a certain reference value for the optimization of MRI images of patients with ovarian endometriosis and for prognostic nursing.

## 2. Materials and Methods

### 2.1. Study Objects

116 patients with ovarian endometriosis in the hospital from February 2018 to April 2021 were chosen as the objects. All the patients underwent an MRI examination. The age of the patients varied at 24–41 years old, with an average age of 33.51 ± 6.76 years old. The average course of the disease for all patients lasted for 8.25 ± 2.47 months, and the course of the disease ranged from 5 to 12 months. All procedures of this study had been approved by the ethics committee of the hospital, and all objects included in the study signed the informed consent forms.

Inclusion criteria: the patients were determined with ovarian endometriosis through pathological diagnosis and accompanied with dysmenorrhea, infertility, menstrual disorders, and other symptoms. The patients had various and complete medical records and signed the informed consent forms. Exclusion criteria: the patients with other malignant tumors, severe cognitive impairment or mental illness, or congenital ovarian hypoplasia were excluded.

### 2.2. Grouping and Nursing Methods

All patients were randomly divided into two groups, with 58 cases in each. One group received routine nursing, which was denoted as the control group; the other group received comprehensive nursing, denoted as the experimental group. Comprehensive nursing consisted of preoperative nursing and postoperative nursing. The focus of preoperative nursing was to communicate with patients timely, ease their emotions, and encourage them to build confidence. Postoperative nursing included a timely recording of the patients' wound condition, cleaning, instructing the patients to perform lower extremity activities to prevent thrombosis, and giving the patients reasonable dietary advice.

### 2.3. MRI Scanning Device and Parameters

The magnetic resonance scanning instrument used in this research was a 3.0T magnetic resonance scanner. All patients (set as the research group) were required to fast for 12 hours before MRI scanning, empty the stool, and fill the bladder properly. They received a certain degree of breathing training before the examination. When MRI scanning was performed, an 18-channel body surface phased array coil was used to cover the patient's pelvis. The external coil was used as the radiofrequency transmitting coil, and the abdominal phased array coil was as the receiving coil, and then the three-dimensional positioning of the patient was made to determine the scope of the scanning. Sagittal and coronal *T*1-weighted imaging (*T*1WI), *T*2-weighted imaging (*T*2WI), presaturated fat-suppressed *T*2-weighted imaging (*T*2WI-FS), and diffusion-weighted imaging (DWI) sequences were performed for scanning. The specific parameters are listed in Table 1.

### 2.4. Lesion Segmentation Model under the Artificial Intelligence FCM Algorithm

To deal with the image recognition issue with noise of traditional MRI, the artificial intelligence FCM algorithm was applied in this study. The flow of the FCM algorithm was described as follows.

First of all, the obtained data were set and classified. It was defined that any element *s* in any domain *S* had only two options, which were classified and not classified to belong to set *B*. The characteristic function of set *B* was expressed as

(1) μsB=0,s∉B1,s∈B.

The value range of *μ*_*B*_(*s*) was {0, 1}. On this basis, the concept of the fuzzy set was introduced, and the expression of the fuzzy set was shown in equation (2). After that, clustering analysis was used to distinguish and classify feature objects efficiently. This was a simple and computationally efficient method, requiring no training process, and had been widely used in pattern recognition, image segmentation, and other fields. When the FCM algorithm was performed, it was necessary to define all the objects *S* to be classified. With the distribution law of the sample in the feature space, *S* was divided into several disjoint subsets based on a specific distance metric, which is shown in equation (3). The subsets needed to meet equations (4) and (5):

(2) s⟶μsB,μsB∈0,1,

(3) $$\begin{array}{c} S=\left\{S_{1},S_{2},\ldots ,S_{n}\right\}\subset R^{sxt}, \end{array}$$

(4) $$\begin{array}{c} S_{1}\cup S_{2}\cup \cdots \cup S_{C}=S, \end{array}$$

(5) $$\begin{array}{c} S_{i}\cap S_{j}=\emptyset ,1\leq i,j\leq c. \end{array}$$

By dividing each element into different categories and iteratively optimizing the results, the affiliation between the record sample and the category was expressed as equation (6). The record sample and the category were of the affiliation matrix of the hard C-means (HCM) clustering algorithm, which had a good classification effect on the data conforming to the hyperellipsoid distribution. The sample set *S* also belonged to equation (4), the total number of elements in the sample set was set as *x*, the element feature value was *z*, and the number of classification categories was *c*. The mean square error was used to measure the classification results of the objective function under the HCM algorithm, and the expression is shown as equation (7).

(6) $$\begin{array}{c} U=\left[\begin{array}{c} \mu_{11,}\mu_{12,}\ldots \mu_{1x,}\\ \mu_{21,}\mu_{22,}\ldots \mu_{2x,}\\ \ldots \\ \mu_{c1,}\mu_{c2,}\ldots \mu_{cx,} \end{array}\right], \end{array}$$

(7) $$\begin{array}{c} J_{\text{HCM}}\left(U,W\right)=\sum_{i=1}^{c}\sum_{g=1}^{x}\mu_{ig}\left\|s_{g}-w_{i}\right\|=\sum_{i=1}^{c}\sum_{g=1}^{x}\mu_{ig}d_{ig}{}^{2}. \end{array}$$

In equation (6), *μ*_*ij*,_1 ≤ *i*, *j* ≤ *c* represented the affiliation of the element *j* to the category *i*, and in equation (7), *w*_*i*_ represented each clustering center and ‖*s*_*g*_ − *w*_*i*_‖ represented a certain paradigm of clustering centers and feature vectors. Afterward, the clustering was completed by seeking the extreme value of the objective function *J*_HCM_(*U*, *W*), and the update function of the affiliation matrix can be derived using the Lagrangian multiplier. This update function could be expressed as equation (8), and the update function of the clustering center could be described as equation (9).

(8) $$\begin{array}{c} \mu_{ig}=\left\{\begin{array}{cc} 1, & \text{if }d_{ij}=\underset{1\leq g\leq c}{\min}\left\{1\leq i\leq c,1\leq j\leq x\right\}\\ 0, & \text{others} \end{array}\right., \end{array}$$

(9) $$\begin{array}{c} w_{i}=\frac{\sum_{j=1}^{x}\mu_{ij}s_{j}}{\sum_{j=1}^{x}\mu_{ij}},1\leq i\leq c. \end{array}$$

For the affiliation matrix of the FCM algorithm, the affiliation between its record sample and its category also met equation (6), which was obtained based on the HCM algorithm. The objective function expression of FCM was

(10) $$\begin{array}{c} J_{\text{FCM}}\left(U,W\right)=\sum_{i=1}^{c}\sum_{g=1}^{x}{\left(\mu_{ig}\right)}^{r}\left\|s_{g}-w_{i}\right\|=\sum_{i=1}^{c}\sum_{g=1}^{x}\mu_{ig}d_{ig}{}^{2}. \end{array}$$

**Figure 1:**
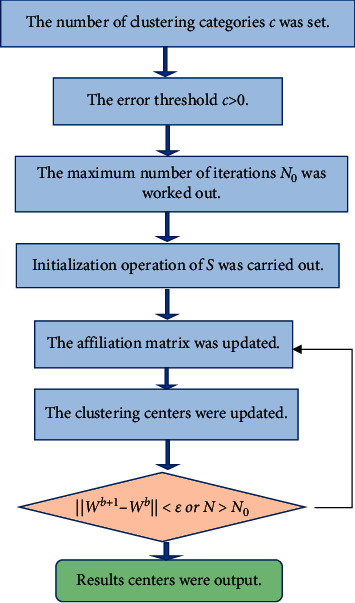
Flowchart of the FCM solution.

In equation (10), *w*_*i*_ stood for each clustering center and ‖*s*_*g*_ − *w*_*i*_‖ was a certain paradigm between clustering centers and feature vectors. *r* was the fuzzy-weighted indicator. As the value of *r* increased, the smoothing effect of clustering on fuzzy affiliation was more obvious, and its value interval was (1.1, 5). After, the clustering was completed by finding the extreme value of the objective function *J*_FCM_(*U*, *W*). The Lagrangian multiplier was applied to derive the update function of the affiliation matrix, which could be expressed as equation (11). The update function of the clustering center was expressed as equation (12).  Figure 1 shows the flowchart of the FCM solution. Thresholding was performed on the optimized fuzzy affiliation matrix of the FCM algorithm, and the thresholding process met equation (13).

(11) $$\begin{array}{c} \mu_{ig}=\left\{\begin{array}{cc} \frac{1}{\sum_{g=1}^{c}{\left(d_{ij}/d_{gj}\right)}^{2/\left(r-1\right)}} & \text{if }d_{ij}>0\\ 1, & \text{others} \end{array}\right., \end{array}$$

(12) $$\begin{array}{c} w_{i}=\frac{\sum_{j=1}^{x}{\left(\mu_{ij}\right)}^{r}s_{j}}{\sum_{j=1}^{x}{\left(\mu_{ij}\right)}^{r}},1\leq i\leq c, \end{array}$$

(13) $$\begin{array}{c} \mu_{ij}=\left\{\begin{array}{cc} 1, & \text{if }\mu_{ij}=\underset{1\leq g\leq c}{\max}\left\{\mu_{gj}\right\}1\leq i\leq c,1\leq j\leq x\\ 0, & \text{others} \end{array}\right.. \end{array}$$

For the pixel classification method, the pixel gray-level feature caused a lot of calculation redundancy when segmenting the medical images by the FCM algorithm, and the histogram information was used to replace it. In the calculation process, the size of the target image was needed to be set as *A* × *B*, and the histogram of the image was recorded as *h*(*g*), *g* = 1, 2,…, *N* − 1. The histogram represented the probability of each gray-level histogram appearing in the image, and *N* represented the total number of gray levels. The FCM objective function after gray-level clustering could be expressed as

(14) $$\begin{array}{c} J_{H}\left(U,W,S\right)=\sum_{g=1}^{N-1}\sum_{i=1}^{c}{\left(\mu_{ig}\right)}^{r}h\left(g\right)\left\|g-w_{i}\right\|. \end{array}$$

In equation (14), *μ*_*ig*_ stood for the fuzzy affiliation of the gray-level *g* to the *i*-th clustering center and ‖*g* − *w*_*i*_‖ was the distance from the gray-level *g* to the *i*-th clustering center. Then, the update function of the clustering center is shown as equation (15), obtained by seeking the extreme value of the improved objective function and the partial derivative of the Lagrangian function.

(15) $$\begin{array}{c} w_{i}=\frac{\sum_{g=1}^{N-1}h\left(g\right){\left(\mu_{ij}\right)}^{r}g}{\sum_{j=1}^{x}h\left(g\right){\left(\mu_{ij}\right)}^{r}},i=1,2,\ldots ,c. \end{array}$$

### 2.5. MRI Image Quality Evaluation under Artificial Intelligence Three-Dimensional FCM Algorithm

In this study, the patients' MRI images were diagnosed by two radiologists. The processing effect of the artificial intelligence algorithm was evaluated by the diagnostic accuracy, Dice, sensitivity, and specificity indicators of the MRI images. The equations to compute the indicators are shown as equations (16), (17), and–(18):

(16) $$\begin{array}{c} \text{Dice}=\frac{|A\cap C|}{|A|+\left|C\right|/2}, \end{array}$$

(17) $$\begin{array}{c} \text{Sensitivity}=\frac{\left|A\cap C\right|}{\left|A\right|}, \end{array}$$

(18) $$\begin{array}{c} \text{Specificity}=\frac{|B\cap D|}{|B|}. \end{array}$$

In the three equations above, *A* and *C* were the real lesion area and the lesion area determined by the artificial intelligence algorithm, respectively, of the ovarian endometriosis. *B* and *D* were the other parts outside the real lesion area and the lesion area under the artificial intelligence algorithm, respectively.

### 2.6. Observation Indicators

The MRI image diagnosis results of ovarian endometriosis and the actual pathological results were compared in this study, by comparing the traditional HCM algorithm and the improved artificial intelligence FCM algorithm. The diagnostic accuracy before and after image processing under the two algorithms were calculated, respectively, which were used to comprehensively evaluate the application value of the improved artificial intelligence FCM algorithm for MRI diagnosis of ovarian endometriosis.

The nursing satisfaction of patients and the incidence of postoperative adverse reactions were used for the evaluation of the nursing effect of patients in the two groups. The nursing satisfaction score >80 was regarded as very satisfied, 61–80 score was regarded as relatively satisfied, score <60 was unsatisfied, and the total number of satisfied cases was the sum of the first two. The common adverse reactions of ovarian endometriosis included infection, abdominal pain, and wound bleeding.

### 2.7. Statistical Methods

The test data were processed by SPSS19.0. The measurement data were expressed by mean ± standard deviation ($\bar{x}$ ± *s*), the comparison of the means between two groups was made by *t*-test, and the enumeration data was expressed by percentage (%) and was tested by the *χ*^2^ test. *P* < 0.05 suggested that the difference was statistically significant.

## 3. Results

### 3.1. Performance Test Results of the Improved Artificial Intelligence FCM Algorithm

**Figure 2:**
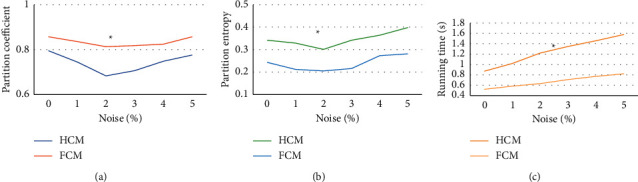
Performance analysis of the two algorithms under the training set. (a–c) Comparison chart of the partition coefficient, partition entropy, and running time of the two algorithms, respectively.  ^*∗*^compared to those of the HCM algorithm, *P* < 0.05.

The partition coefficient, partition entropy, and iteration number of the two algorithms are shown in Figure 2, which were obtained in the training set test under different noise conditions. The results showed that when the noise was 0%, 1%, 2%, 3%, 4%, and 5%, the partition coefficient of the traditional HCM algorithm was 0.7951, 0.7451, 0.683, 0.706, 0.748, and 0.776, respectively; the partition entropy was 0.342, 0.329, 0.301, 0.342, 0.364, and 0.398, respectively; and the running time was 0.87, 1.02, 1.22, 1.35, 1.46, and 1.58s, respectively. Meanwhile, for the improved artificial intelligence FCM algorithm, the partition coefficient was 0.857, 0.836, 0.813, 0.818, 0.824, and 0.857; respectively; the partition entropy was 0.243, 0.212, 0.205, 0.216, 0.273, and 0.281, respectively; and the running time was 0.52, 0.58, 0.63, 0.71, 0.77, and 0.82 s, respectively. It was found that, compared with those of the traditional HCM algorithm, the partition coefficients under each noise of the improved artificial intelligence FCM algorithm were significantly higher, and the partition entropy and running time of the FCM algorithm were significantly lower; the differences were statistically significant (*P* < 0.05).

### 3.2. General Information of Patients

**Figure 3:**
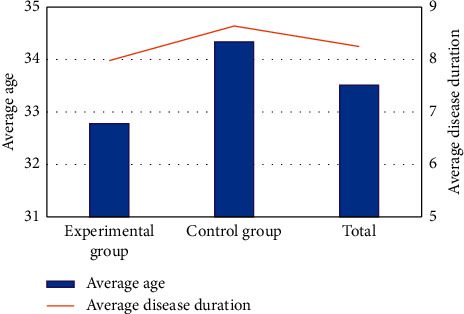
Summary chart of patients' average age and course of the disease.

Figure 3 is a summary chart of the average age of the patients and their courses of the disease. It was observed that the average age of all patients was 33.51 ± 6.76 years old, and the average course of the disease was 8.25 ± 2.47 months. For the patients in the experimental group, the average age was 32.78 ± 7.86 years old, and the average course of the disease was 7.98 ± 5.39 months. For patients in the control group, the average age of them was 34.34 ± 9.55 years old, and the average course of the disease lasted for 8.64 ± 6.12 months. There was no significant difference in the patients' average age and the average course of the disease between the two groups, without a statistical significance (*P* < 0.05).

**Figure 4:**
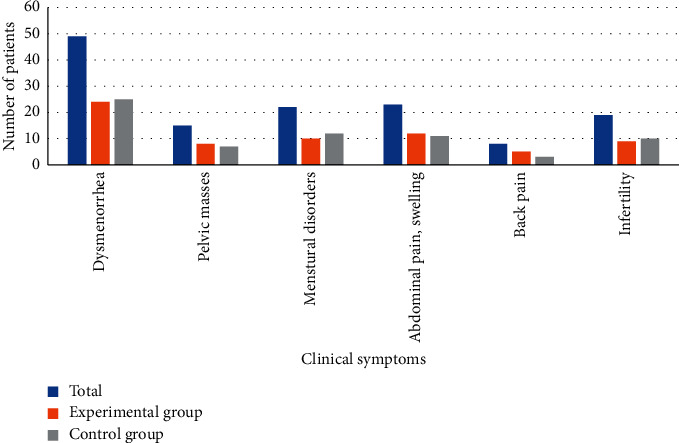
Clinical symptom distribution of the patients.

Figure 4 shows the distribution of clinical symptoms of the patients. Among the 116 patients with ovarian endometriosis, there were 49 patients with dysmenorrhea, 15 patients with pelvic masses, 22 patients with menstrual disorders, 23 patients with abdominal pain and swelling, 23 patients with low back pain, and 19 patients with infertility. The differences in the number of patients with the above symptoms were not significant between the experimental group and the control group, and so it was not statistically significant (*P* > 0.05).

### 3.3. MRI Image Analysis under the Improved Artificial Intelligence FCM Algorithm

**Figure 5:**
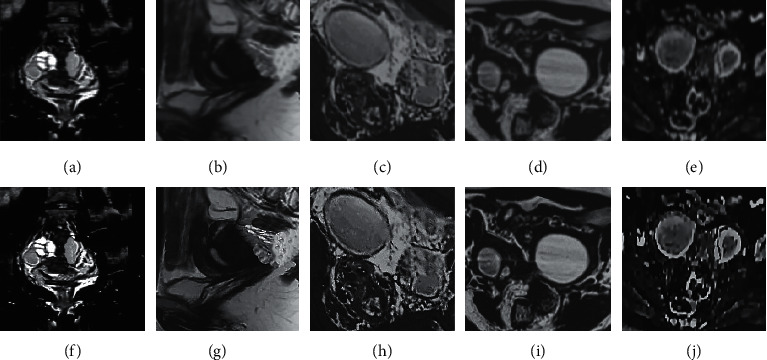
MRI images before and after processing by the artificial intelligence algorithm. (a–e) Traditional abdominal MRI images of coronal presaturated T2WI-FS, sagittal T2WI, sagittal presaturated T2WI-FS, sagittal T1WI, and sagittal DWI, respectively. (f–j) Abdominal MRI images processed by the improved artificial intelligence FCM algorithm of coronal presaturated T2WI-FS, sagittal T2WI, sagittal presaturated T2WI-FS, and sagittal T1WI, and sagittal DWI, respectively.

Figure 5 shows the MRI images before and after processing by the artificial intelligence FCM algorithm, including that of coronal presaturated T2WI-FS, sagittal T2WI, sagittal presaturated T2WI-FS, sagittal T1WI, and sagittal DWI. From Figure 5, the clarity and the recognition performance for the lesions of the MRI images were significantly improved as the images were processed by the artificial intelligence FCM algorithm. Compared with the MRI images before processing, the processed images showed clearer structures of the uterus, adjacent organs, pelvic wall, and lymph nodes in the transverse position, and the sagittal images could show the positional relationship between the lesions and the vagina, bladder, and rectum more intuitively.

### 3.4. MRI Image Quality under the Improved Artificial Intelligence FCM Algorithm

**Figure 6:**
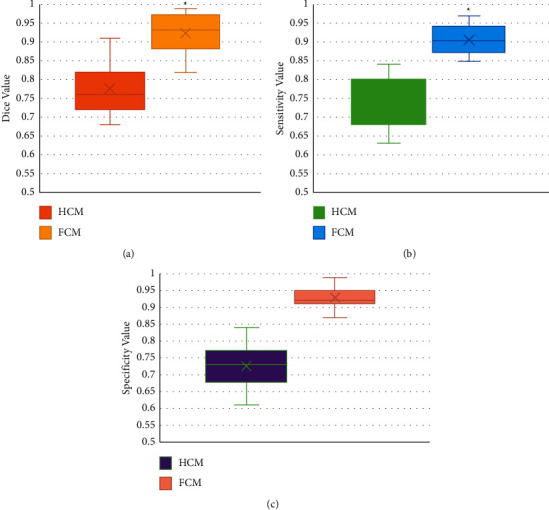
Comparison charts of image quality evaluation indicators under different algorithms. (a–c) Comparison charts of dice, sensitivity, and specificity, respectively.  ^*∗*^Compared to those under the HCM algorithm, *P* < 0.05.

The diagnostic indicators of Dice, sensitivity, and specificity were compared as the MRI images were processed by the traditional HCM algorithm and the improved artificial intelligence FCM algorithm, respectively. The results obtained are shown in Figure 6. For the MRI images of ovarian endometriosis, the average values of Dice, sensitivity, and specificity processed by the traditional HCM algorithm were 0.77, 0.73, and 0.72, respectively; and those processed by the improved artificial intelligence FCM algorithm were 0.92, 0.90, and 0.93, respectively; the differences in varied indicators between the two algorithms were statistically significant (*P* < 0.05).

### 3.5. Evaluation of Diagnostic Effect with MRI Images under the Improved Artificial Intelligence FCM Algorithm

**Figure 7:**
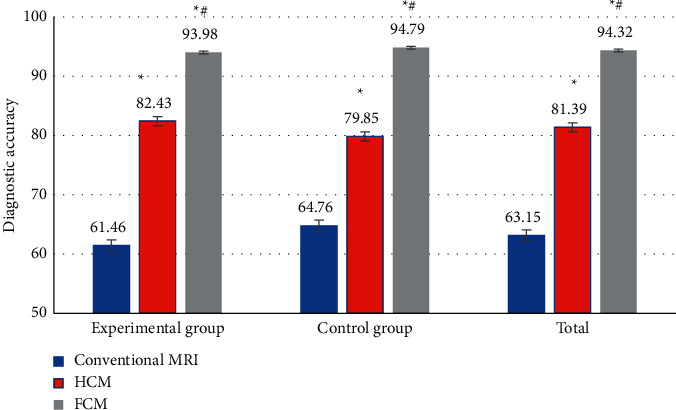
Comparison of the imaging diagnostic accuracy under different algorithms.  ^*∗*^Compared to that under conventional MRI, *P* < 0.05; ^#^compared to that under the HCM algorithm, *P* < 0.05.

The diagnostic accuracies under different algorithms are shown in Figure 7. The diagnostic accuracy of MRI image diagnosis for all patients was counted as 63.15 ± 4.12%, 81.39 ± 3.11%, and 94.32 ± 3.05%, respectively, as the images were processed by the conventional multimodal MRI, the traditional HCM algorithm, and the improved artificial intelligence FCM algorithm. The diagnostic accuracy in the experimental group was 61.46 ± 3.56%, 79.85 ± 3.68%, and 94.79 ± 3.21%, respectively, under the three methods. It was shown that the diagnostic accuracies under the latter two algorithms were significantly higher than that under the conventional MRI (*P* < 0.05), and the diagnostic accuracy under the improved artificial intelligence FCM algorithm was also significantly higher than that under the traditional HCM algorithm (*P* < 0.05).

### 3.6. Evaluation of Nursing Satisfaction and Incidence of Adverse Reactions in the Two Groups

**Figure 8:**
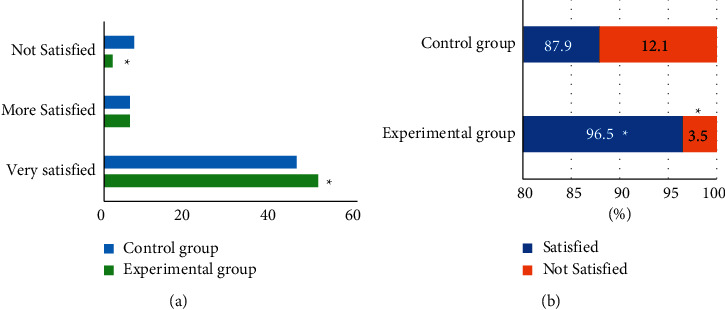
Comparison of imaging diagnostic accuracy under different algorithms. (a) Comparison chart of the nursing satisfaction of the two groups; (b) The overall nursing satisfaction percentage of the two groups.  ^*∗*^Compared to the data of the control group, *P* < 0.05.

The nursing satisfaction of patients in the two groups are shown in Figure 8. It can be observed that, in the nursing satisfaction survey, the number of very satisfied patients in the experimental group was significantly more than that in the control group, and the number of unsatisfied patients was significantly less than that in the control group, with the statistically significant differences (*P* < 0.05). The overall nursing satisfaction of patients in the experimental group was 96.5%, and that in the control group was 87.9%; the difference was statistically significant.

**Figure 9:**
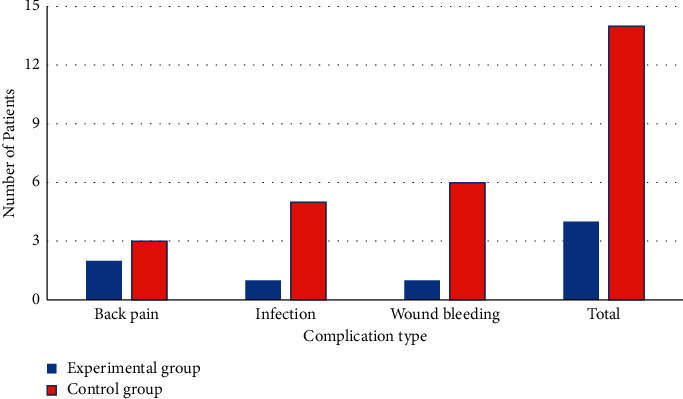
The incidences of adverse reactions of patients in different groups.

The incidences of adverse reactions of patients in the two groups are shown in Figure 9. It was shown that the incidence of adverse reactions in the experimental group was significantly lower than that in the control group, with a significant difference (*P* < 0.05).

## 4. Discussion

With some technical issues in imaging examination methods currently, there would be some misdiagnosis in the clinical practice of many diseases [15]. For the reduction of deviation in the imaging examination as much as possible, and the improvement of the imaging quality, various computer-assisted diagnosis and treatment methods came into being [16]. FCM algorithm has been used in the intelligent processing of medical images increasingly [17]. In recent years, it is reported that the FCM has been fully applied in the intelligent processing of ultrasound, CT, MRI, and other medical images, and the involved diseases include brain tumor, lung cancer, liver cancer, breast cancer, and gastric cancer [18, 19].

In this research, an artificial intelligence FCM clustering algorithm was designed for the MRI image characteristics of patients with ovarian endometriosis, and it was applied for the clinical MRI diagnosis of ovarian endometriosis. Compared with the traditional HCM algorithm, the improved artificial intelligence FCM algorithm had significantly higher segmentation coefficients under various noises. The segmentation entropy and running time of the processing by this algorithm were highly reduced, and the differences were of statistical significance (*P* < 0.05). The clarity of MRI images processed by the algorithm and the identification performance of lesions were greatly improved as well. Hua et al. [20] utilized an improved multiview FCM clustering algorithm (IMV-FCM) to improve the segmentation accuracy of the algorithm for brain images. In this case, brain tissues could be accurately segmented in the actual segmentation results of a large number of brain MRI images. Compared with several related clustering algorithms, the IMV-FCM algorithm has a better adaptability and better clustering performance.

The average values of Dice, sensitivity, and specificity of MRI images of ovarian endometriosis were 0.77, 0.73, and 0.72, respectively, processed by the traditional HCM algorithm. Those processed by the improved artificial intelligence FCM algorithm were 0.92, 0.90, and 0.93, respectively. Compared with the traditional algorithm, the values of these 3 indicators were remarkably improved, and the differences were statistically significant (*P* < 0.05). In addition, the diagnostic accuracy of the improved artificial intelligence FCM algorithm was 94.32 ± 3.05%, which was also significantly higher than the 81.39 ± 3.11% of the traditional HCM algorithm (*P* < 0.05). Jin and Chang [21] confirmed the diagnostic value of the optimized FCM algorithm combined with coronal MRI in the diagnosis of tracheal foreign bodies in children. MRI images had a higher distribution coefficient, lower segmentation entropy, larger interclass similarity, and better image segmentation effect for coronal MRI images. The diagnostic rate of tracheal foreign bodies in children was significantly promoted. This is consistent with the research results of Su et al. [22]. But the optimization procedures related to the improved artificial intelligence FCM algorithm are very complex, so further in-depth research is needed.

Similarly, the overall nursing satisfaction of patients in the experimental group was 96.5% in this study, while that in the control group was 87.9%, with a statistically significant difference (*P* < 0.05). The incidence of postoperative adverse reactions in the experimental group was significantly lower than that in the control group, and the difference was significant (*P* < 0.05). Thus, it was confirmed that, compared with conventional nursing, comprehensive nursing could improve the nursing satisfaction of patients and, meanwhile, reduce the incidence of postoperative adverse reactions.

## 5. Conclusion

In this study, for the MRI image characteristics of patients with ovarian endometriosis, the artificial intelligence FCM algorithm was applied in the MRI diagnosis of ovarian endometriosis clinically. As results found, the recognition and segmentation of lesions were significantly improved in the multimodal MRI images based on the artificial intelligence FCM algorithm, which could significantly increase the clinical diagnosis accuracy of ovarian endometriosis. However, there were still some shortcomings in this study. In the discussion of the fusion processing of multimodal MRI images, a FCM image fusion scheme had not been found to highlight the characteristics of MRI images. Besides, the FCM algorithm in this study had a poor processing effect on the segmentation edge of the lesions, which needed to be optimized. Altogether, the MRI under the artificial intelligence FCM algorithm could improve the accuracy of clinical diagnosis of ovarian endometriosis significantly, which brought a certain reference value for the efficiency improvement of clinical diagnosis and treatment of ovarian endometriosis.

## Figures and Tables

**Table 1:** MRI scanning parameters.

Parameters	Sagittal plane	Coronal plane
Time of echo	85–95 ms	8–11 ms
Time of repetition	3800–4100 ms	500–600 ms
Echo train length	17	3
Number of excitations	4	2
Layer thickness	6 mm	6 mm
Interlayer spacing	1 mm	1 mm
Matrix	512 × 512	512 × 512

## Data Availability

The data used to support the findings of this study are available from the corresponding author upon request.

## References

[1] Bulun S. E., Wan Y., Matei D. (2019). Epithelial mutations in endometriosis: link to ovarian cancer. *Endocrinology*.

[2] Liu N., Ren Q. (2021). Magnetic resonance imaging feature analysis and evaluation of tubal patency under convolutional neural network in the diagnosis of infertility. *Contrast Media and Molecular Imaging*.

[3] Samartzis E. P., Labidi-Galy S. I., Moschetta M. (2020). Endometriosis-associated ovarian carcinomas: insights into pathogenesis, diagnostics, and therapeutic targets-a narrative review. *Annals of Translational Medicine*.

[4] Vercellini P., Viganò P., Buggio L. (2018). Perimenopausal management of ovarian endometriosis and associated cancer risk: when is medical or surgical treatment indicated?. *Best Practice & Research Clinical Obstetrics & Gynaecology*.

[5] Somigliana E., Viganò P., Benaglia L., Busnelli A., Paffoni A., Vercellini P. (2019). Ovarian stimulation and endometriosis progression or recurrence: a systematic review. *Reproductive BioMedicine Online*.

[6] Rolla E. (2019). Endometriosis: advances and controversies in classification, pathogenesis, diagnosis, and treatment. *F1000Research*.

[7] Wang Y., Nicholes K., Shih I.-M. (2020). The origin and pathogenesis of endometriosis. *Annual Review of Pathology: Mechanisms of Disease*.

[8] Nisenblat V., Bossuyt P. M. M., Farquhar C., Johnson N., Hull M. L. (2016). Imaging modalities for the non-invasive diagnosis of endometriosis. *Cochrane Database of Systematic Reviews*.

[9] Piessens S., Edwards A. (2020). Sonographic evaluation for endometriosis in routine pelvic ultrasound. *Journal of Minimally Invasive Gynecology*.

[10] Foti P. V., Farina R., Palmucci S. (2018). Endometriosis: clinical features, MR imaging findings and pathologic correlation. *Insights into Imaging*.

[11] Lee H. J. (2020). Usefulness of subtraction pelvic magnetic resonance imaging for detection of ovarian endometriosis. *Yeungnam University Journal of Medicine*.

[12] Hu M., Zhong Y., Xie S., Lv H., Lv Z. (2021). Fuzzy system based medical image processing for brain disease prediction. *Frontiers in Neuroscience*.

[13] Li Y., Zhao J., Lv Z., Li J. (2021). Medical image fusion method by deep learning. *International Journal of Cognitive Computing in Engineering*.

[14] Yu Z., Amin S. U., Alhussein M., Lv Z. (2021). Research on disease prediction based on improved DeepFM and IoMT. *IEEE Access*.

[15] Knott K. D., Seraphim A., Augusto J. B. (2020). The prognostic significance of quantitative myocardial perfusion: an artificial intelligence based approach using perfusion mapping. *Circulation*.

[16] Gates E. D. H., Lin J. S., Weinberg J. S. (2020). Imaging-based algorithm for the local grading of glioma. *American Journal of Neuroradiology*.

[17] Albizu A., Fang R., Indahlastari A. (2020). Machine learning and individual variability in electric field characteristics predict tDCS treatment response. *Brain Stimulation*.

[18] Papp L., Spielvogel C. P., Grubmüller B. (2021). Supervised machine learning enables non-invasive lesion characterization in primary prostate cancer with [68Ga]Ga-PSMA-11 PET/MRI. *European Journal of Nuclear Medicine and Molecular Imaging*.

[19] Pereira S., Pinto A., Alves V., Silva C. A. (2016). Brain tumor segmentation using convolutional neural networks in MRI images. *IEEE Transactions on Medical Imaging*.

[20] Hua L., Gu Y., Gu X., Xue J., Ni T. (2021). A novel brain MRI image segmentation method using an improved multi-view fuzzy c-means clustering algorithm. *Frontiers in Neuroscience*.

[21] Jin L., Chang K. (2021). Optimized fuzzy C-means algorithm-based coronal magnetic resonance imaging scanning in tracheal foreign bodies of children. *Journal of Healthcare Engineering*.

[22] Su J. H., Thomas F. T., Kasoff W. S. (2019). Thalamus Optimized Multi Atlas Segmentation (THOMAS): fast, fully automated segmentation of thalamic nuclei from structural MRI. *NeuroImage*.

